# Supplementary material for: Investigating online activity in UK adolescent mental health patients: a feasibility study using a natural language processing approach for electronic health records
Source: BMJ Open. 2023 May 25;13(5):e061640. doi: 10.1136/bmjopen-2022-061640 (PMC10230886; doi:10.1136/bmjopen-2022-061640)
Supplement: Supplementary data [file bmjopen-2022-061640supp001.pdf]

APPENDIX A: Annotation guidelines for adolescents Online Activity in CRIS

Introduction

This document contains the annotation guidelines for annotating clinician mentions of online activity in clinical text. We have broadly grouped online activity into groups: social media, internet, and online gaming, though there is some overlap. The aim is to be able to automatically identify clinician mentions of these factors documented in mental health records to then investigate associations between these exposures and self-harm outcomes in adolescents.

General Annotating

- You will need to read each document from start to finish for relevant mentions; which will then be highlighted.
- Where possible annotate only the relevant word(s).
- In some cases, a larger section of annotation will be required- annotate as much as is required to give context.
- All annotations should be given a class and an attribute from the below options.
- Each individual annotation can have only one of each.
- Not all mentions are explicit, we will accept inferred mentions if you can ascertain meaning from the context, further guidance below.

SOCIAL MEDIA
INTERNET
ONLINE GAMING

Class Annotating

Social Media

We are interested in patterns and the nature of social media use. Social media is for these guidelines defined as: *websites and applications that enable users to create and share content or to participate in social networking.* Mentions may refer to specific platforms included in the gazetteer such as: Instagram, Twitter, Facebook, Snapchat, or to a behaviours.

Examples: "Chatting to their friends online", "Talking to friends online"

Internet

We are interested in patterns and the nature of internet use and content viewed or shared online.

Examples:"… spends a lot of time online"

Online Gaming

We are interested in online gaming and have included general terms and more specific titles of games commonly used within the timeframe of our dataset.

Example: "Spends a lot of time playing video games"
Example: "Playing games on the internet"

Other online use

Since social media and internet activity are often accessed via mobile devices we have also included: *iPhone, iPad, Blackberry, Smartphone.* Where there is suggestion that these are used for online gaming or social media annotate accordingly if the exact use is not clear annotate as INTERNET.

Sedgwick R, *et al. BMJ Open* 2023; 13:e061640. doi: 10.1136/bmjopen-2022-061640

Specific platforms

Pinterest
YouTube

These can have social media-like functions but are more commonly viewed as internet platforms and should therefore be annotated as INTERNET.

Attribute Annotating

Classification:
- DETRIMENTAL
- NEUTRAL
- OTHER

Supportive_Category:
- OTHER
- SUPPORTIVE_CARER
- SUPPORTIVE_PROFESSIONAL_ADVICE
- SUPPORTIVE_YOUNG_PERSON

Detrimental

This attribute is for mentions which appear to be having a negative impact on the young person, either directly (such as via bullying) or indirectly (restricting other activities, causing arguments, affecting sleep).

Examples:
"playing computer games most of the time rather than continuing with his work"
"sleep is disturbed as z spends a great deal of time at home on the computer"
"received a message on Facebook from her best friend saying that their friendship was fake"

Neutral

Neutral attributes are where it is not possible from the text to determine the context or sufficient detail to ascribe a detrimental or supportive label.

Examples:
"does not go on Facebook or chat rooms"
"z is spending more time on the computer"

Supportive

SUPPORTIVE_CARER
This attribute is used for mentions which appear to relate to advice or support that the carer is receiving, likely in relation to the young person's condition.
Example: "mum reports finding the National Autistic Society parent forum a helpful resource"

SUPPORTIVE_PROFESSIONAL_ADVICE
This would be relevant if a clinician has recommended or signposted the young person or carer to resources online.
Example: "I gave mum the details of the National Autistic Society website"

SUPPORTIVE_YOUNG_PERSON
This could be because a digital resource has been specifically helpful. In addition, we classify mentions of a child 'liking' or 'enjoying' am activity as supportive. The rationale for this is that for young people with mental health difficulties enjoyable activities are often incorporated therapeutically.

Sedgwick R, *et al. BMJ Open* 2023; 13:e061640. doi: 10.1136/bmjopen-2022-061640

Example: "z and his friend are making and editing Minecraft videos to upload to YouTube and he reports that this makes him feel happy".
Example: "z found a useful anti-bullying website"
Example: "z relaxes watching videos on YoutTube"

Additional refinements

When annotating highlight as much of the sentence/paragraph required to understand the context of the mention.

If it is clear that a document is a copy/paste version of one in the same corpus (this may be that an event has been copied to create a letter, or subtly different versions of the same letter have been sent to different parties) these should be annotated consistently.

Ambiguous examples:

Sometimes there are mentions which are ambiguous or appear to suggest contradictory qualities.

If context was creating a website this could be internet (i.e. YouTube), if for social networking site/sharing with contacts (i.e. Instagram) might be social media.

Example: "...posting images/photos online"

With no additional context we do not know what exact activity this is referring to (could be non-web-based, web browsing, online gaming, or social media).

Example: " enjoys playing on the computer"
Example: "He loves playing on the computer, but mum is worried that this stopping him socialising or doing normal activities with the family"

Clusters of mentions

Two mentions of different classes written one after the other can be coded separately.

*Example: "she likes playing **video games** and watching **videos online**".*
video games ONLINE GAMING>SUPPORTIVE_YP
videos online INTERNET>SUPPORTIVE_YP.

*Example: "Spends a lot of time playing **video games** like **Fortnite**"*
video games ONLINE GAMING>NEUTRAL
fortnite ONLINE GAMING>NEUTRAL

*Example: "She is being bullied via **Facebook, Instagram and Snapchat**"*
Facebook SOCIAL MEDIA>DETRIMENTAL
Instagram SOCIAL MEDIA>DETRIMENTAL
Snapchat SOCIAL MEDIA>DETRIMENTAL

*Example**: "**Z loves to read, watch movies or TV clips on **YouTube** and play **Sims"***
YouTube INTERNET>SUPPORTIVE_YP
Sims ONLINE GAMING>SUPPORTIVE_YP

Negated mentions

Negation detection

"...does not use social media"
"...has not been bullied on Facebook"
"...avoiding the internet as this was not helpful and adding stress"
"...now playing video games less"

Sedgwick R, *et al. BMJ Open* 2023; 13:e061640. doi: 10.1136/bmjopen-2022-061640
