# Supplementary material for: Investigating online activity in UK adolescent mental health patients: a feasibility study using a natural language processing approach for electronic health records
Source: BMJ Open. 2023 May 25;13(5):e061640. doi: 10.1136/bmjopen-2022-061640 (PMC10230886; doi:10.1136/bmjopen-2022-061640)
Supplement: Author's manuscript [file bmjopen-2022-061640.draft_revisions.pdf]

BMJ Open

BMJ Open is committed to open peer review. As part of this commitment we make the peer review history of every article we publish publicly available.

When an article is published we post the peer reviewers' comments and the authors' responses online. We also post the versions of the paper that were used during peer review. These are the versions that the peer review comments apply to.

The versions of the paper that follow are the versions that were submitted during the peer review process. They are not the versions of record or the final published versions. They should not be cited or distributed as the published version of this manuscript.

BMJ Open is an open access journal and the full, final, typeset and author-corrected version of record of the manuscript is available on our site with no access controls, subscription charges or pay-per-view fees (http://bmjopen.bmj.com).

If you have any questions on BMJ Open's open peer review process please email info.bmjopen@bmj.com

BMJ Open

Online activity in adolescent mental health patients: A Natural Language Processing approach for Electronic Health Records

Journal:	*BMJ Open*
Manuscript ID	bmjopen-2022-061640
Article Type:	Original research
Date Submitted by the Author:	10-Feb-2022
Complete List of Authors:	Sedgwick, Rosemary; King's College London; South London and Maudsley NHS Foundation Trust<br>Bittar, André; King's College London<br>Kalsi, Herkiran; King's College London<br>Barack, Tamara; King's College London<br>Downs, Johnny; King's College London, Institute of Psychiatry, Psychology and Neuroscience; South London and Maudsley Mental Health NHS Trust<br>Dutta, Rina; King's College London, Institute of Psychiatry, Psychology and Neuroscience; South London and Maudsley Mental Health NHS Trust
Keywords:	Child & adolescent psychiatry < PSYCHIATRY, Health informatics < BIOTECHNOLOGY & BIOINFORMATICS, World Wide Web technology < BIOTECHNOLOGY & BIOINFORMATICS

SCHOLARONE™
Manuscripts

I, the Submitting Author has the right to grant and does grant on behalf of all authors of the Work (as defined in the below author licence), an exclusive licence and/or a non-exclusive licence for contributions from authors who are: i) UK Crown employees; ii) where BMJ has agreed a CC-BY licence shall apply, and/or iii) in accordance with the terms applicable for US Federal Government officers or employees acting as part of their official duties; on a worldwide, perpetual, irrevocable, royalty-free basis to BMJ Publishing Group Ltd ("BMJ") its licensees and where the relevant Journal is co-owned by BMJ to the co-owners of the Journal, to publish the Work in this journal and any other BMJ products and to exploit all rights, as set out in our licence.

The Submitting Author accepts and understands that any supply made under these terms is made by BMJ to the Submitting Author unless you are acting as an employee on behalf of your employer or a postgraduate student of an affiliated institution which is paying any applicable article publishing charge ("APC") for Open Access articles. Where the Submitting Author wishes to make the Work available on an Open Access basis (and intends to pay the relevant APC), the terms of reuse of such Open Access shall be governed by a Creative Commons licence – details of these licences and which Creative Commons licence will apply to this Work are set out in our licence referred to above.

Other than as permitted in any relevant BMJ Author's Self Archiving Policies, I confirm this Work has not been accepted for publication elsewhere, is not being considered for publication elsewhere and does not duplicate material already published. I confirm all authors consent to publication of this Work and authorise the granting of this licence.

For peer review only

Online activity in adolescent mental health patients: A Natural Language Processing approach for Electronic Health Records

R Sedgwick[1,2], A Bittar[1], H Kalsi[1], T Barack[1], J Downs[1,2], *R Dutta[1,2]

[1]Institute of Psychiatry, Psychology and Neuroscience, King's College London

[2]South London and Maudsley NHS Foundation Trust

*Corresponding author: rina.dutta@kcl.ac.uk

For peer review only

ABSTRACT

Objectives: To assess the feasibility of using a Natural Language Processing (NLP) application for extraction of free text online activity mentions in adolescent mental health patient Electronic Health Records (EHRs).

Setting: The Clinical Records Interactive Search (CRIS) system allows detailed research based on de-identified EHRs from the South London and Maudsley NHS Foundation Trust (SLaM), a large south London Mental Health Trust providing secondary and tertiary mental health care.

Participants and Methods: We developed a gazetteer of online activity terms and annotation guidelines, from 5,480 clinical notes (200 adolescents, aged 11-17 years) receiving specialist mental health care. The pre-processing and manual curation steps of this real-world dataset allowed development of a rule-based NLP application to automate identification of online activity (internet, social media, online gaming) mentions in EHRs. The context of each mention was also recorded manually as: supportive, detrimental or neutral for additional analysis.

Results: The NLP application performed with good precision (0.97) and recall (0.94). Preliminary analyses found 34% of online activity mentions were considered to have been documented within a supportive context for the young person, 38% detrimental and 28% 'neutral'.

Conclusion: Our results provide an important example of a rule-based NLP methodology to accurately identify online activity recording in EHRS, enabling researchers to now investigate associations with a range of adolescent mental health outcomes.

For peer review only

STRENGTHS AND LIMITATIONS OF THIS STUDY

- Investigation of online activity, such as internet browsing, social media and online gaming, is increasingly being acknowledged as a new research area of interest to mental health professionals, with potentially detrimental exposures and supportive interventions requiring further study.
- Natural Language Processing can be used to detect novel clinical risks within Electronic Health Records, but no detailed methodology is available on how online activity can be identified from clinical notes for epidemiological analyses. To the authors knowledge this

For peer review only - http://bmjopen.bmj.com/site/about/guidelines.xhtml

paper is the first of its kind to describe the feasibility and development of an NLP application for extraction of online activity mentions in EHRs for use in research.

- Recording of online activity in free text EHRs will be dependent on both patient report and the detail of documentation by clinicians, and therefore may not represent the full extent of young people's online use.
- Information extracted using the methods outlined in this paper could provide valuable avenues for further research into the recorded online activity of young adolescent mental health patients and associations with mental health outcomes.

BACKGROUND

Use of the internet, social media and online gaming are now ubiquitous amongst Children and young people (CYP). There are general concerns about the potentially harmful impact of screentime on children and young adolescents health, and particularly their mental health [1]. There are also some more established, specific risks online, such as cyberbullying [2]. Internet use is associated with a wide range of adverse outcomes such as self-harm and suicidal behaviour [3 4], disordered eating and body image issues [5], and symptoms of Attention Deficit Hyperactivity Disorder (ADHD) [6]. Problematic video-gaming and social media are also associated with several health issues, such as conduct problems and sedentary behavior [7]. In addition, there is growing evidence, beyond mental health research, for associations between technology and being overweight or obese [8], with poorer academic performance [9] and exacerbation of educational inequalities [10].

Internet Gaming Disorder was added to the fifth Diagnostic Statistical Manual (DSM-5) [11] and Gaming Disorder added to the International Classification of Diseases (ICD-11) [12]. Age, gender, personality characteristics and parental behaviour may all influence adolescents' choice of games [13] and gaming can be done via a number of different devices, both online and offline. Digital platforms are commonly used by adolescents and a wealth of information may be shared online. There are now consensus recommendations that asking about online activities should be part of routine clinical assessments [14-16]. The prevalence with which these are noted in mental health assessments completed in Child and Adolescent Mental Health Services (CAMHS), and the context in which they are

For peer review only

recorded, has not been studied to date. The existing evidence for the impact of online activity on CYP is predominantly from cross-sectional survey data and often includes minimal detail about online activities, often with a focus on amount of use, or defined by terms such as Problematic Internet Use (PIU) [17]. Given the wide range of social media platforms, devices, games, and content on the internet it will be important to gain a more nuanced and real-world understanding of what adolescents are engaging with online. Studying a clinical population of mental health patients will highlight which disorders may predispose adolescents to negative psychological and social impacts of online activity, but also what they find supportive. This study provides valuable contextualising information about the recording of online activity in clinical encounters with adolescent mental health patients.

There are validated measures for smartphone, internet and gaming addiction, the most commonly used being the Smartphone Addiction Scale (SAS) [18], Internet Addiction Test (IAT) and the Chen's Internet Addiction Scale (CIAS) [19], but these are not widely used by clinicians within the UK and there is significant heterogeneity within the research literature. As these structured scales are not commonly used in clinical practice, they will not be uploaded within structured fields on Electronic Health Records (EHRs). However, in the UK 83% of children aged 12-15 have a smartphone and 69% have at least one social media profile [20] and adolescents with mental disorders spend more time online than those without a mental disorder [21]. CYP may not show symptoms suggestive of behavioral addiction, but this does not mean that they are not engaging in activities that may be harmful.

As part of mental health assessment and follow-up, clinicians will often discuss the adolescent's interests and how they spend their time, as well as triggers to recent episodes or relapses, such as cyberbullying. The EHRs therefore contain unstructured free text data about online activity of CYP in contact with CAMHS. Advances in health informatics mean that information extraction tools can be used to automate the extraction of such information to allow computation to be done on the previously unstructured data using Natural Language Processing (NLP). This approach has already created opportunities to analyse large textual datasets and can now accurately detect mentions of other complex phenomena such as suicidality [22-25] and obsessive compulsive symptoms [26]. This study seeks to answer the question of whether an NLP application can derive information on the similarly

For peer review only - http://bmjopen.bmj.com/site/about/guidelines.xhtml

complex and broad construct of adolescent mental health patient online activity. This will have implication for researchers wishing to undertake large scale epidemiological research, as well as clinicians who could use this personalised data to inform patient care.

METHODS

Data Source

The Clinical Records Interactive Search (CRIS) system allows detailed research based on EHRs from the South London and Maudsley NHS Foundation Trust (SLaM), a large south London Mental Health Trust providing secondary and tertiary care to residents of Southwark, Lambeth, Lewisham and Croydon [27]. The use of CRIS data for research was approved by the Oxfordshire Research Ethics Committee C (reference 08/H0606/71 + 5). CRIS data is used by researchers in a de-identified and data-secure format and patients have the choice to opt-out of their data being used. CRIS approval for this project has been granted by the CRIS oversight committee (Project reference: 18-102) and all data for use in this research has been accessed in accordance with CRIS Governance procedures. Care may be provided in mental health settings such as clinics or psychiatric hospitals, or in acute health settings such as emergency departments. In 2014 there were 250,000 million documents [27]. As of September 2019, the EHRs of over 350,000 patients, including over 5.7 million text documents can be analysed. Clinicians may enter clinical information in a variety of different sections within the EHRs, including: 'events' (unstructured notes), forms (i.e. risk assessment), or attached clinical documents such as letters. Events and letters are most commonly used to record clinical information and sometimes the same information may be duplicated across locations.

Clinical Cohort and Corpus Development

In order to develop an NLP application, it was necessary to generate an adolescent data set within CRIS. Event and attachment documents (n=1,601,422) were derived from 23,455 adolescent patients aged 11 to 17 in contact with CAMHS between 31/04/2009 and 31/03/2016, as described by Velupillai et al [28]. As illustrated in figure 1, from this, a corpus of documents was extracted from a randomly selected group of 200 patients who had a number of EHR documents within the 1st and 3rd

For peer review only - http://bmjopen.bmj.com/site/about/guidelines.xhtml

quartiles (document n=5,480). This ensured that patients with particularly high or low numbers of records were excluded, as these patients were less likely to be representative of the general clinical population accessing CAMHS, either due to high intensity of contact (such as with prolonged inpatient care) or lack of contact due to non-engagement. Diagnosis was not used as an inclusion or exclusion criterion.

Mentions of online activity in EHRs

Key word searches were a basic but necessary first step to establish prevalence and variability of such terms within free text, especially for such a rapidly evolving and broad construct as online activity. Available literature was searched until there was a saturation of terms. The search included published work, grey literature publications online and policy documents, supported by consultation with adolescents through local patient advisory groups up to 2019. This formed the basis of the gazetteer of terms, developed to convey topics that included online devices (i.e., computer, iPad), internet terms (e.g., websites, specific sites (e.g., YouTube), online games (e.g., Fortnite), social media terms (e.g., forum*) and specific platforms including Facebook, Twitter, and Instagram. The full gazetteer used for the final stages of this research is available in Table 1.

Table 1: Online activity gazetteer

**Social Media**	**Internet**	**Online Gaming**
#	Android	Call of Duty
4chan	Blackberry	Club Penguin
askFM	Computer	Computer gam*
Bebo	Dark Web	Computer-gam*
Blog*	Deep web	Coraline
Chatroom*	Googl*	Counter strike
cyber-bully*	Internet	Dota 2
cyberbully*	iphone	Dragon age
e-communi*	Laptop	Fallout
Face book	Mobile phone	Game Boy
Facebook	Online	Game-boy

For peer review only - http://bmjopen.bmj.com/site/about/guidelines.xhtml

FB	PC	Gaming
Flickr	Pinterest	Ghostbusters
Forum*	Skype	Grand Theft Auto
Hashtag*	Smartphone	HALO
Image Sharing	surf* the web	League of legends
Instagram	web address	Minecraft
Instant messag*	web brows*	Miniclip
Linkedin	web surfing	Nintendo
lolcow	web-brows*	Online Gam*
Myspace	web-surfing	PC gam*
Periscope	website*	Playstation
Recovery account	you tub*	PS3
Reddit	youtub*	PS4
Snapchat	ipad	Sims
Social Media	i-pad	Smite
Social Network*		video game
Spam Account		Wii
Tumblr		World of tanks
Tweet*		World of warcraft
Twitter		Xbox
Video sharing		X-box
Vimeo		Xmen
WhatsApp		X-men
Wordpress		Fortnite
		Pokemon
		Fortnight
		DS

For peer review only

Extracting EHRs for manual curation and pre-processing

The clinical corpus from the inception cohort of 200 patients was used for all subsequent analysis and development. Based on the rationale that a varied lexicon would be used to describe online media use, the gazetteer of key terms was used to identify and filter documents from the corpus with at least one

For peer review only - http://bmjopen.bmj.com/site/about/guidelines.xhtml

of the search terms, to avoid reading a large volume of unrelated documents. By applying this filter, we identified 217 documents containing at least one of the terms, from 84/200 patients. These were used to gain further contextual insight and identify additional terms relevant to the concepts, including any common misspellings or abbreviations found (i.e. Face Book, FB). Documents with one or more terms from the gazetteer were analysed in detail by two researchers (RS and HK). Many documents were found to be irrelevant 'noise'. Examples were disclaimer messages at the bottom of email contacts, or use of the NHS Trust website in letter headers. The term 'email' was found to be generating too much noise for inclusion. Decisions such as this were agreed during regular consensus meetings with the research group.

Developing manual coding rules

To ensure that future research could be targeted towards more specific exposures it was necessary to split the search terms to represent three separate classes of mention: internet, social media, and online gaming. The manual curation also identified broad sentiment attributes within clinician documentation: Detrimental, Supportive or Neutral. For example, mention of Facebook in the context of bullying and a subsequent presentation to hospital would be coded as 'SOCIAL MEDIA_DETRIMENTAL'. During the initial scoping exercise supportive mentions were further split into sub-categories to allow for more detailed future analysis and to better capture the context of mentions in the text. This included online activity that adolescents have referred to as supportive, clinician offered supportive advice (e.g. recommending online resources) and online activity which supports carers (e.g. use of a mental health support forum). Annotation guidelines were developed for the above class and attribute rules to facilitate consistent manual annotation by more than one researcher.

Manual annotation of Online Activity and sentiment attributes in EHRs

The pre-processing steps, when applied to the EHRs of the inception cohort, yielded a development corpus of 200 documents from the overall 5480 (derived from 89 of the 200 patients), which formed the dataset for the pilot analysis reported below in results. The corpus of 200 documents was divided

For peer review only - http://bmjopen.bmj.com/site/about/guidelines.xhtml

and annotated for class and attributes by two researchers (RS and HK) using the annotation guidelines. Thirty documents were double annotated and there was an inter-annotator agreement of kappa coefficient=0.91 for class, 0.68 for attributes and 0.94 for supportive category [29].

Development of the Online Activity NLP application

The pre-processing steps outlined above paved the way for development of the NLP application, designed to automate identification of mentions of online activity use in EHRs. During the manual annotation (human-rater) stage, contextualising online activity raised some challenges. The sentiment attributes were found to be heterogeneous, often lacking detail and more subject to human inter-rater disagreement. Therefore, the algorithm was developed for automation of the class of mention (internet, social media, or online gaming), based on the manual coding rules applied to the development corpus. Further details and examples can be found in the Annotation Guidelines, Appendix A.

The Online Activity NLP application is a rule-based system based on the spaCy NLP library for Python (version 2.1.3). The application uses four levels of processing, applied sequentially to each document:

1. *Text cleaning:* removal of "unwanted" document sections by regular expression replacement.
2. *Linguistic pre-processing:* sentence and word tokenisation, lemmatisation, and part-of-speech tagging.
3. *Lexical annotation:* terms in the text are tagged according to the gazetteer (e.g. 'computer', 'website' are tagged as INTERNET, 'cyberbully*', 'forum' and 'Instagram' are tagged as SOCIAL_MEDIA) and 'Fortnite' and 'online gaming' are tagged as ONLINE_GAMING.
4. *Token sequence annotation:* sequences of tokens (i.e. words) are annotated and classified (e.g. the pattern '(chat|communicat|talk).* online' is tagged as SOCIAL_MEDIA, '(play|playing) fortnite' is tagged as ONLINE_GAMING, etc. This step also removes annotations ("untags") from mentions that were erroneously tagged in the lexical annotation step.

For peer review only - http://bmjopen.bmj.com/site/about/guidelines.xhtml

Patient and public involvement

Development of the gazetteer of online activity terms was supported by consultation with adolescent mental health patients through local patient advisory groups up to 2019.

RESULTS

The development corpus (n=200) documents extracted through the pre-processing steps (each document containing at least one term from the gazetteer) contained N=243 individual mentions of online activity. Duplicate and irrelevant mentions were removed (N=115). The remaining 101 documents (64 patients) contained 128 mentions of internet (N=64), social media (N=32), online gaming (N=32). Mean age was 14 (range 11-17 years), 37 males and 27 females, with ethnicity representative of the local population.

Contextualising mentions of online activity

There were in total, 44 supportive mentions (34%), 48 detrimental mentions (38%), 36 neutral mentions (28%). No 'other' mentions were recorded in this development corpus. Supportive mentions were sub-divided into supportive for the young person (N=25), where a clinician was offering supportive advice (N=17) or where a carer had reported an online activity as helpful (N=2). Each class was also analysed independently to provide pilot data on these different exposures. Internet mentions were 33% detrimental, 48% supportive, 19% neutral. Social media mentions were predominantly female and classed as detrimental (50%), with little supportive benefits (9%). Online gaming was predominantly amongst male users and showed detrimental (34%), supportive (31%) and neutral (34%) context.

Evaluation of the Online Activity NLP application

An evaluation corpus was curated using EHRs from an expanded date range of the inception cohort, from CRIS origination to 02/07/2019. These adolescents were 11-17 at the time of presentation

For peer review only - http://bmjopen.bmj.com/site/about/guidelines.xhtml

(between 2009-16), therefore it was anticipated that not all records (n= 12795) would be relevant. As the research group were interested in the CAMHS population specifically, only documents pertaining to adolescents who were still age 18 or younger at the time of documentation were included. Records of less than 50 words were removed due to the lack of relevance. Documents included in the development corpus were also removed (n=200). The remaining evaluation corpus (n=5972) was randomly divided between two researchers (RS and TB) and all relevant mentions of Internet/Social media/Online gaming were manually annotated according to the annotation guidelines. Attributes were again manually annotated. To establish the human inter-rater agreement, 200 documents, both with and without annotations were double annotated, yielding a kappa coefficient of 0.94 for annotation of class (internet, social media, online gaming). Following a consensus discussion, discrepancies were resolved, and adjudicated documents were included to produce a 'gold standard' Evaluation Corpus containing 535 individual annotations, from 5972 documents. This evaluation revealed a precision of 0.97 and recall of 0.94 and kappa=0.91. Span agreement showed a precision of 0.69 and recall 0.80, with full results available in Table 2.

**Table 2: Performance of the Online Activity NLP application on the Evaluation Corpus (n=5972)**		
**Evaluation results**		
	**Span agreement**	**Class (Internet, Social media, [illegible]**
**Precision (macro)**	0.69	0.97
**Recall (macro)**	0.80	0.94
**F-score (macro)**	0.74	0.95
**Precision (micro)**	N/A	0.95
**Recall (micro)**	N/A	0.95
**F-score (micro)**	N/A	0.95
**Kappa**	N/A	0.91

For peer review only

DISCUSSION

This study provides evidence of the feasibility of using free-text EHR data for the evaluation of online activity in mental health patients and to the authors knowledge is the first of its kind to use this methodology. The use of digital interventions in mental health is rapidly growing and there is interest in how these developments should be evaluated in future. In the meantime, it is vital that more evidence-based guidelines reach clinicians to ensure the quality of documentation facilitates research into this important and timely area. This is a move supported by the UK Royal College of Psychiatrists in their 2020 report, which calls for urgent funding of high-quality, longitudinal research into the effects of technology on the mental health of young people, and the need for technology companies to provide user-generated data for research [16].

Clinician mentions of online activity will be influenced by the clinician's personal knowledge and experience. The results of our scoping exercises revealed that despite guidance from the British Psychological Association [15] and Royal College of Psychiatrists [14] the detail of documentation has historically been poor. This is however likely to improve, given increasing acknowledgement of the important role of digital technology and mental health for CYP. As more mental health applications and online resources are recommended by clinicians; familiarity will increase, these discussions will more frequently take place between patients and professionals at CAMHS and recording in free-text EHRs will improve.

The focus of NLP development within CRIS has historically been on symptoms, but detection of behaviours and activities CYP engage in is also required if we are to better understand the impact on mental health. Automated detection of cyberbullying has been attempted in social media text [30], and a bullying NLP application has been developed [31]. The NLP application we outline here is important as it will be able to capture emerging online activities and behaviours in the EHRs of CYP, providing opportunities for much needed longitudinal research into online activity and mental health and wellbeing, which to date has been lacking. These developments can inform our understanding of the specific risks and benefits of online exposures; inform clinical guidelines and help target future interventions caused by digital exposures and to evaluate interventions delivered through these platforms.

For peer review only - http://bmjopen.bmj.com/site/about/guidelines.xhtml

There are no similar studies available for direct comparison and therefore the strengths of the NLP application are encouraging. It has shown good precision (0.97) and recall (0.94) in automating detection of mentions of internet, social media, and online gaming in our corpus of clinical notes, enabling research into online activities within a large adolescent mental health population. We divided online activity into broad classes: internet, social media, and online gaming, though it is worth noting that there is increasing overlap. We found that broadly social media was reported in the EHRs as more harmful than online gaming, which may be an important hypothesis generating finding. The concepts of 'supportive', 'detrimental' and 'neutral', have been shown to be promising attributes for automation and incorporation into future iterations of the online activity NLP application. However, limitations to human-coder agreement requires further work and a more nuanced taxonomy of terms may be required to accurately reflect the online activity of CYP.

There are limitations to the clinical interpretation possible from our data. The pilot data reported in this paper included any mention of enjoyment as 'supportive' for the young person, unless there was any negating information. Identification and nurturing of enjoyable activities and hobbies can be a useful tool when working with adolescent patients in CAMHS. But, there is also the possibility of these becoming excessive and having a 'detrimental' impact, especially given the increasing concern about gaming disorder [11 32]. Perspectives of the young person and a carer may differ, but this may not be documented by the clinician. It will also be necessary in later iterations of the NLP application to incorporate negating terms and phrases, as well as greater sensitivity for which subject (CYP themselves or third party) the mention of online activity relates to. This and other nuances will become increasingly important on more contemporary datasets, though it is worth noting that it was not considered a major limitation in the historical corpus reported here.

There are limitations to the rule-based approach with such a rapidly evolving field. New games, social media platforms, websites and apps will be hard to keep up with and omission of these titles from the gazetteer may bias studies towards certain online activities. The gazetteer can be added to and amended based on the context of the data and emergence of new popular terms, but this will require a degree of vigilance from users wishing to apply it to contemporary datasets, or those with other groups, such as adults or disorder-specific cohorts. The application displayed insufficient

For peer review only - http://bmjopen.bmj.com/site/about/guidelines.xhtml

contextual disambiguation for the following words: computer, Internet, mobile phone, online, PC, website. It performed less well distinguishing class from longer spans of free text i.e., *playing games with friends online* or *playing games on the computer* being incorrectly labelled 'internet' rather than 'online gaming'. Mention of all specific websites described in CRIS would not be feasible, but inclusion of *www.,.co.uk* or other more generic identifiers resulted in too many false positives. Similarly, '*email*'* generated too many false positives during development to be included. These may therefore be false negatives that should be considered when using the NLP application and it is possible that in some circumstance's precision could be sacrificed for greater recall.

This paper outlines the developments in NLP for use in EHR's within CRIS, but the Online Activity NLP application could also be adapted for other clinical data sets to allow reproduction of results. User-generated data could be another application for this NLP approach and may more accurately capture adolescent online behaviour, unhampered by the recall and reporting bias associated with self-report questionnaires or discussion with a clinician. NLP can already be applied to risk assessment of self-injurious behaviour in user-generated content [33] and the Linguistic Inquiry and Word Count (LIWC) has shown promise in assessing emotional wellbeing from Facebook posts [34]. Social media data has potential as a rich data source for identification of medical and mental health conditions [35 36] and with further refinement, discussion of risk-associated online activity or 'online harms' [37] could be another avenue for the application of NLP, such as that outlined here. These advancements could eventually lead to earlier detection of at-risk adolescents and targeting of interventions, a field already developing in suicide prevention [38].

There are also potential clinical applications for this work when applied to EHRs. The app could be valuable for characterising online activity patterns for specific patient groups, such as those with eating disorders or Autism Spectrum Disorder, and the impact on later recovery. Since the Covid-19 pandemic, young people's online activity has accelerated, with greater reliance on online means of communication, education, and access to mental health support. Our NLP application could provide valuable insight into these trends; providing information on an individual and epidemiological level to guide recommendations. There is also potential for adaptation to more dynamic uses, such as EHR surveillance to track the burden of adverse online experiences through established methods such

For peer review only - http://bmjopen.bmj.com/site/about/guidelines.xhtml

as Audit and feedback, which can result in important improvements in clinical practice[39]. Information such as this, presented in accessible ways such as clinician dashboards, could support rapid synthesis of risk factors within an individual or across a service and identify areas of unmet need and potential treatment targets.

CONCLUSION

We have developed a highly accurate Online Activity NLP application for use in EHRs, which can incorporate keywords as online platforms and services develop over time. This will allow further research using CRIS data to investigate novel risk factor research into a range of adolescent mental health outcomes. It also opens the door for EHR surveillance and clinical monitoring, enabling clinicians to track the burden of adverse online experiences at an individual and service level. Beyond its proven utilisation in EHRs, tools such as this also have the potential to be adapted to other clinical or non-clinical datasets, which could enhance our understanding of these new phenomena and the impact on adolescent health and wellbeing.

DATA AVAILABILITY

The data accessed by CRIS remain within an NHS firewall and governance is provided by a patient-led oversight committee. Access to data is restricted to honorary or substantive employees of the South London and Maudsley NHS Foundation Trust and governed by a local oversight committee who review and approve applications to extract and analyse data for research. Subject to these conditions, data access is encouraged and those interested should contact the CRIS academic lead.

ACKNOWLEDGMENTS

The authors acknowledge infrastructure support from the National Institute for Health Research (NIHR). The views expressed are those of the author(s) and not necessarily those of the NHS, the NIHR or the Department of Health and Social Care.

AUTHOR CONTRIBUTIONS

For peer review only - http://bmjopen.bmj.com/site/about/guidelines.xhtml

Rosemary Sedgwick developed the concept, and led on the study design, data collection, writing of results and final draft of the manuscript. Andre Bittar developed the NLP application and ran the evaluations. Manual coding rules were written by Rosemary Sedgwick with input from the other authors, including Johnny Downs and Rina Dutta. Reviewing of EHRs for the manual curation stage was performed by Rosemary Sedgwick and Herkiran Kalsi. Reviewing the EHRs for the Evaluation Corpus was performed by Rosemary Sedgwick and Tamara Barack. All authors were involved in the writing and review of the manuscript. All authors declare no competing conflict of interest.

FUNDING SOURCES

RS was funded by an National Institute for Health Research (NIHR) Academic Clinical Fellowship and a NIHR Biomedical Research Centre at South London and Maudsley NHS Foundation Trust Preparatory Fellowship.

JD is supported by an NIHR Clinician Science Fellowship award and has received support from a Medical Research Council (MRC) Clinical Research Training Fellowship and Psychiatry Research Trust Peggy Pollak Research Fellowship in Developmental Psychiatry.

RD is funded by a Clinician Scientist Fellowship from the Health Foundation in partnership with the Academy of Medical Sciences and her work is supported by the NIHR Biomedical Research Centre at South London and Maudsley NHS Foundation Trust and King's College London.

For peer review only

COMPETING INTERESTS

RD declares previous research funding received from Janssen.

The remaining authors declare that the research was conducted in the absence of any commercial or financial relationships that could be construed as a potential conflict of interest.

REFERENCES

1. Stiglic N, Viner RM. Effects of screentime on the health and well-being of children and adolescents: a systematic review of reviews. *BMJ Open* 2019;9(1):e023191. doi: 10.1136/bmjopen-2018-023191

2. John A, Glendenning AC, Marchant A, et al. Self-Harm, Suicidal Behaviours, and Cyberbullying in Children and Young People: Systematic Review. *Journal of Medical Internet Research* 2018;20(4):e129. doi: 10.2196/jmir.9044
3. Marchant A, Hawton K, Stewart A, et al. A systematic review of the relationship between internet use, self-harm and suicidal behaviour in young people: The good, the bad and the unknown. *PLoS One* 2017;12(8):e0181722. doi: doi: 10.1371/journal.pone.0181722.
4. Sedgwick R, Epstein S, Dutta R, et al. Social media, internet use and suicide attempts in adolescents. LID - 10.1097/YCO.0000000000000547 [doi]. *Curr Opin Psychiatry* 2019(1473-6578 (Electronic))
5. Holland G, Tiggemann M. A systematic review of the impact of the use of social networking sites on body image and disordered eating outcomes. *Body Image* 2016;17:100-10. doi: https://doi.org/10.1016/j.bodyim.2016.02.008
6. Ra CK, Cho J, Stone MD, et al. Association of Digital Media Use With Subsequent Symptoms of Attention-Deficit/Hyperactivity Disorder Among Adolescents. *JAMA* 2018;320(3):255-63. doi: 10.1001/jama.2018.8931
7. Merelle SYM, Kleiboer AM, Schotanus M, et al. Which health-related problems are associated with problematic video-gaming or social media use in adolescents? A large-scale cross-sectional study. *Clinical Neuropsychiatry* 2017;14(1):11-19.
8. Herodotou C. Young children and tablets: A systematic review of effects on learning and development. *Journal of Computer Assisted Learning* 2018;34(1):1-9. doi: 10.1111/jcal.12220
9. Sampasa-Kanyinga H, Chaput J-P, Hamilton HA. Social Media Use, School Connectedness, and Academic Performance Among Adolescents. *The Journal of Primary Prevention* 2019;40(2):189-211. doi: 10.1007/s10935-019-00543-6
10. Performance CfE. Ill Communication: Technology, distraction & student performance Discussion Paper No 1350, 2015.
11. Arlington V. Diagnostic and Statistical Manual of Mental Disorders, Fifth Edition.: American Psychiatric Association 2013.
12. Office for national statistics. 2018. Internet access- households and individuals: 2018 2018 [Available from: https://www.ons.gov.uk/peoplepopulationandcommunity/householdcharacteristics/homeinternetandsocialmediausage/bulletins/internetaccesshouseholdsandindividuals/2018 accessed 06/09/2018 2018.
13. John N, Sharma MK, Kapanee ARM. Gaming- a bane or a boon-a systematic review. *Asian J Psychiatry* 2019;42:12-17.
14. Psychiatrists RCo. Child & Adolescent Psychiatry Curriculum: January 2018 amendments, 2018.
15. Bristow F, Roberts A. Top tips for working with children, young people and their families: Supporting trainees and other pre-qualified clinicians. *British Psychological Society* 2015
16. RCPsych. Technology use and the mental health of children and young people. 2020;College Report CR225
17. Spada MM. An overview of problematic Internet use. *Addictive Behaviors* 2014;39(1):3-6. doi: https://doi.org/10.1016/j.addbeh.2013.09.007
18. Kwon M, Kim D-J, Cho H, et al. The Smartphone Addiction Scale: Development and Validation of a Short Version for Adolescents. *PLOS ONE* 2014;8(12):e83558. doi: 10.1371/journal.pone.0083558
19. Costa S, Kuss DJ. Current diagnostic procedures and interventions for Gaming Disorders: A Systematic Review. *Front Psychol* 2019;10:578-78. doi: 10.3389/fpsyg.2019.00578
20. Ofcom. Children and Parents: Media Use and Attitudes Report 2018. Ofcom, 2019.
21. Digital N. Mental Health of Children and Young People in England, 2017, 2018.
22. Polling C, Tulloch A, Banerjee S, et al. Using routine clinical and administrative data to produce a dataset of attendances at Emergency Departments following self-harm. *BMC Emerg Med* 2015;15(15):015-0041.
23. Fernandes AC, Dutta R, Velupillai S, et al. Identifying Suicide Ideation and Suicidal Attempts in a Psychiatric Clinical Research Database using Natural Language Processing. *Scientific Reports* 2018;8:7426. doi: 10.1038/s41598-018-25773-2

For peer review only - http://bmjopen.bmj.com/site/about/guidelines.xhtml

24. Downs J, Velupillai S, Gkotsis G, et al. Detection of Suicidality in Adolescents with Autism Spectrum Disorders: Developing a Natural Language Processing Approach for Use in Electronic Health Records. *AMIA Annual Symposium Proceedings* 2017:641-49.
25. Velupillai S, Hadlaczky G, Baca-Garcia E, et al. Risk Assessment Tools and Data-Driven Approaches for Predicting and Preventing Suicidal Behavior. *Frontiers in Psychiatry* 2019(1664-0640 (Print))
26. Chandran D, Robbins DA, Chang C-K, et al. Use of Natural Language Processing to identify Obsessive Compulsive Symptoms in patients with schizophrenia, schizoaffective disorder or bipolar disorder. *Scientific Reports* 2019;9(1):14146. doi: 10.1038/s41598-019-49165-2
27. Perera G, Broadbent M, Callard F, et al. Cohort profile of the South London and Maudsley NHS Foundation Trust Biomedical Research Centre (SLaM BRC) Case Register: current status and recent enhancement of an Electronic Mental Health Record-derived data resource. *BMJ Open* 2016;6(3):e008721. doi: 10.1136/bmjopen-2015-008721
28. Velupillai S, Epstein S, Bittar A, et al. Identifying Suicidal Adolescents from Mental Health Records Using Natural Language Processing. *Studies in health technology and informatics* 2019;264:413-17. doi: 10.3233/SHTI190254
29. Cohen J. A Coefficient of Agreement for Nominal Scales. *Educational and Psychological Measurement* 1960;20(1):37-46. doi: 10.1177/001316446002000104
30. Van Hee C, Jacobs G, Emmery C, et al. Automatic detection of cyberbullying in social media text. *PloS one* 2018;13(10):e0203794-e94. doi: 10.1371/journal.pone.0203794
31. Holden RM, Joanne; Mcgowan, John; Sanyal, Jyoti, Kikoler, Maxim; Simonoff, Emily; Velupillai, Sumithra ; Downs, Johnny. Investigating Bullying as a predictor of Suicidality in a Clinical Sample of Adolescents with Autism Spectrum Disorder. *Autism Research* 2020 doi: (in press, 2020, DOI: 10.1002/aur.2292)
32. International statistical classification of diseases and related health problems (11th Revision): World Health Organization; 2018 [Available from: https://icd.who.int/browse11/l-m/en#/http://id.who.int/icd/entity/1448597234 accessed 7/03/2019.
33. Franz PJ, Nook EC, Mair P, et al. Using Topic Modeling to Detect and Describe Self-Injurious and Related Content on a Large-Scale Digital Platform. LID - 10.1111/sltb.12569 [doi]. *Suicide & life-threatening behavior* 2019(1943-278X (Electronic)) doi: https://doi.org/10.1111/sltb.12569
34. Settanni M, Marengo D. Sharing feelings online: studying emotional well-being via automated text analysis of Facebook posts. *Front Psychol* 2015;6:1045-45. doi: 10.3389/fpsyg.2015.01045
35. Thorstad R, Wolff P. Predicting future mental illness from social media: A big-data approach. *Behav Res Methods* 2019;51(4):1586-600.
36. Merchant RMA-Ohoo, Asch DA, Crutchley PA-Ohoo, et al. Evaluating the predictability of medical conditions from social media posts. *PLoS One* 2019;14(6) doi: doi: 10.1371/journal.pone.0215476
37. Government H. Online Harms White Paper, 2019.
38. Lopez-Castroman JA-Ohoo, Moulahi B, Aze J, et al. Mining social networks to improve suicide prevention: A scoping review. LID - 10.1002/jnr.24404 [doi]. *Journal of Neuroscience Research* 2019(1097-4547 (Electronic)) doi: https://doi.org/10.1002/jnr.24404
39. Ivers N, Jamtvedt G Fau - Flottorp S, Flottorp S Fau - Young JM, et al. Audit and feedback: effects on professional practice and healthcare outcomes. *Cochrane Database Syst Rev* 2012;6(1469-493X (Electronic))

FIGURE LEGENDS

Figure 1: Method for developing a rule-based NLP application for Online Activity. n= number of documents. N=number of mentions.

For peer review only - http://bmjopen.bmj.com/site/about/guidelines.xhtml

Figure 1: Method for developing a rule-based NLP application for Online Activity. n= number of documents. N=number of mentions.

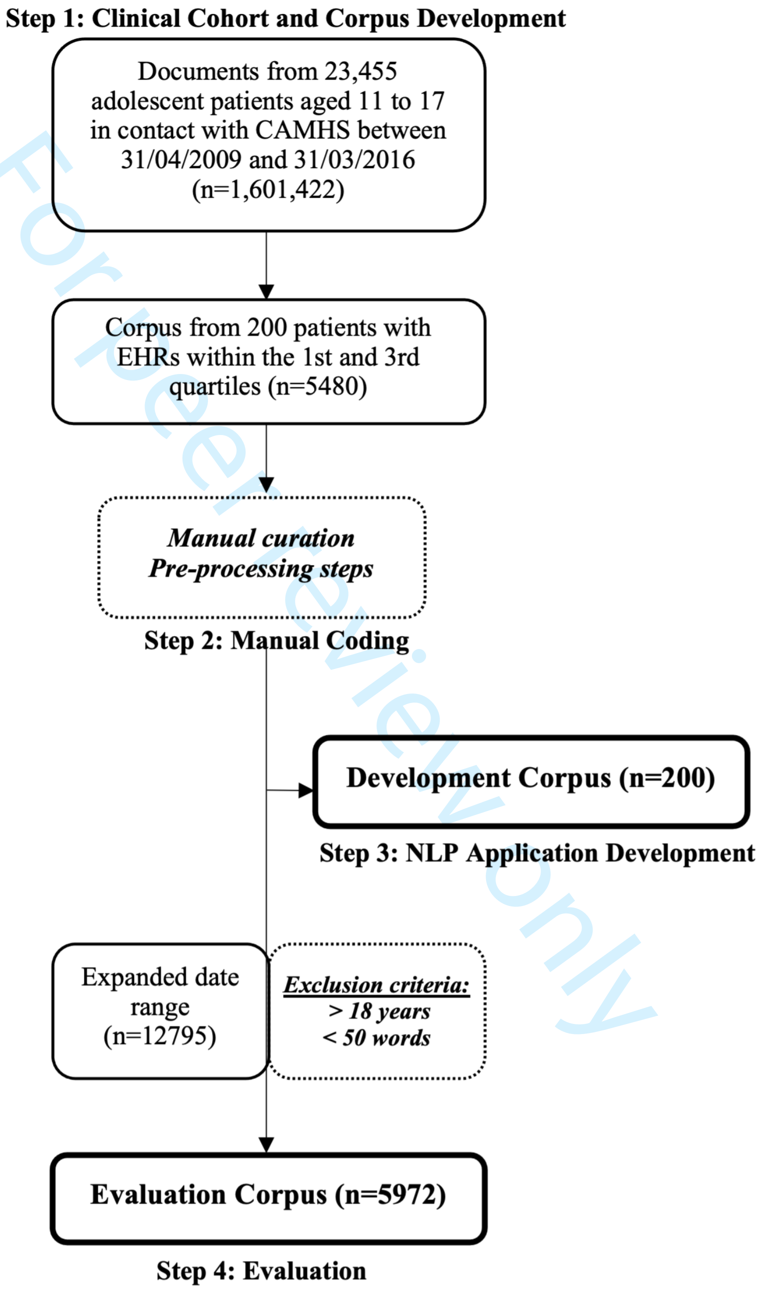

For peer review only

APPENDIX A: Annotation guidelines for adolescents Online Activity in CRIS

Introduction

This document contains the annotation guidelines for annotating clinician mentions of online activity in clinical text. We have broadly grouped online activity into groups: social media, internet, and online gaming, though there is some overlap. The aim is to be able to automatically identify clinician mentions of these factors documented in mental health records to then investigate associations between these exposures and self-harm outcomes in adolescents.

General Annotating

- You will need to read each document from start to finish for relevant mentions; which will then be highlighted.
- Where possible annotate only the relevant word(s).
- In some cases, a larger section of annotation will be required- annotate as much as is required to give context.
- All annotations should be given a class and an attribute from the below options.
- Each individual annotation can have only one of each.
- Not all mentions are explicit, we will accept inferred mentions if you can ascertain meaning from the context, further guidance below.

SOCIAL MEDIA<br>INTERNET<br>ONLINE GAMING

Class Annotating

Social Media

We are interested in patterns and the nature of social media use. Social media is for these guidelines defined as: *websites and applications that enable users to create and share content or to participate in social networking.* Mentions may refer to specific platforms included in the gazetteer such as: Instagram, Twitter, Facebook, Snapchat, or to a behaviours.

Examples: "Chatting to their friends online", "Talking to friends online"

Internet

We are interested in patterns and the nature of internet use and content viewed or shared online.

Examples:"… spends a lot of time online"

Online Gaming

We are interested in online gaming and have included general terms and more specific titles of games commonly used within the timeframe of our dataset.

Example: "Spends a lot of time playing video games"
Example: "Playing games on the internet"

Other online use

Since social media and internet activity are often accessed via mobile devices we have also included: *iPhone*, *iPad*, *Blackberry*, *Smartphone*. Where there is suggestion that these are used for online gaming or social media annotate accordingly if the exact use is not clear annotate as INTERNET.

For peer review only - http://bmjopen.bmj.com/site/about/guidelines.xhtml

Specific platforms

Pinterest
YouTube

These can have social media-like functions but are more commonly viewed as internet platforms and should therefore be annotated as INTERNET.

Attribute Annotating

Classification:<br>DETRIMENTAL<br>NEUTRAL<br>OTHER<br><br>Supportive_Category:<br>OTHER<br>SUPPORTIVE_CARER<br>SUPPORTIVE_PROFESSIONAL_ADVICE<br>SUPPORTIVE_YOUNG_PERSON

Detrimental

This attribute is for mentions which appear to be having a negative impact on the young person, either directly (such as via bullying) or indirectly (restricting other activities, causing arguments, affecting sleep).

Examples:
"playing computer games most of the time rather than continuing with his work"
"sleep is disturbed as z spends a great deal of time at home on the computer"
"received a message on Facebook from her best friend saying that their friendship was fake"

Neutral

Neutral attributes are where it is not possible from the text to determine the context or sufficient detail to ascribe a detrimental or supportive label.

Examples:
"does not go on Facebook or chat rooms"
"z is spending more time on the computer"

Supportive

SUPPORTIVE_CARER

This attribute is used for mentions which appear to relate to advice or support that the carer is receiving, likely in relation to the young person's condition.

Example: "mum reports finding the National Autistic Society parent forum a helpful resource"

SUPPORTIVE_PROFESSIONAL_ADVICE

This would be relevant if a clinician has recommended or signposted the young person or carer to resources online.

Example: "I gave mum the details of the National Autistic Society website"

SUPPORTIVE_YOUNG_PERSON

This could be because a digital resource has been specifically helpful. In addition, we classify mentions of a child 'liking' or 'enjoying' am activity as supportive. The rationale for this is that for young people with mental health difficulties enjoyable activities are often incorporated therapeutically.

For peer review only - http://bmjopen.bmj.com/site/about/guidelines.xhtml

Example: "z and his friend are making and editing Minecraft videos to upload to YouTube and he reports that this makes him feel happy".
Example: "z found a useful anti-bullying website"
Example: "z relaxes watching videos on YoutTube"

Additional refinements

When annotating highlight as much of the sentence/paragraph required to understand the context of the mention.

If it is clear that a document is a copy/paste version of one in the same corpus (this may be that an event has been copied to create a letter, or subtly different versions of the same letter have been sent to different parties) these should be annotated consistently.

Ambiguous examples:

Sometimes there are mentions which are ambiguous or appear to suggest contradictory qualities.

If context was creating a website this could be internet (i.e. YouTube), if for social networking site/sharing with contacts (i.e. Instagram) might be social media.

Example: "...posting images/photos online"

With no additional context we do not know what exact activity this is referring to (could be non-web-based, web browsing, online gaming, or social media).

Example: " enjoys playing on the computer"
Example: "He loves playing on the computer, but mum is worried that this stopping him socialising or doing normal activities with the family"

Clusters of mentions

Two mentions of different classes written one after the other can be coded separately.

*Example: "she likes playing **video games** and watching **videos online**".*
video games ONLINE GAMING>SUPPORTIVE_YP
videos online INTERNET>SUPPORTIVE_YP.

*Example: "Spends a lot of time playing **video games** like **Fortnite**"*
video games ONLINE GAMING>NEUTRAL
fortnite ONLINE GAMING>NEUTRAL

*Example: "She is being bullied via **Facebook, Instagram and Snapchat**"*
Facebook SOCIAL MEDIA>DETRIMENTAL
Instagram SOCIAL MEDIA>DETRIMENTAL
Snapchat SOCIAL MEDIA>DETRIMENTAL

*Example**: "**Z loves to read, watch movies or TV clips on **YouTube** and play **Sims"***
YouTube INTERNET>SUPPORTIVE_YP
Sims ONLINE GAMING>SUPPORTIVE_YP

Negated mentions

Negation detection

"...does not use social media"
"...has not been bullied on Facebook"
"...avoiding the internet as this was not helpful and adding stress"
"...now playing video games less"

For peer review only

BMJ Open

Investigating online activity in UK adolescent mental health patients: a feasibility study using a Natural Language Processing approach for Electronic Health Records

Journal:	*BMJ Open*
Manuscript ID	bmjopen-2022-061640.R1
Article Type:	Original research
Date Submitted by the Author:	31-Mar-2023
Complete List of Authors:	Sedgwick, Rosemary; King's College London; South London and Maudsley NHS Foundation Trust<br>Bittar, André; King's College London<br>Kalsi, Herkiran; King's College London<br>Barack, Tamara; King's College London<br>Downs, Johnny; King's College London, Institute of Psychiatry, Psychology and Neuroscience; South London and Maudsley Mental Health NHS Trust<br>Dutta, Rina; King's College London, Institute of Psychiatry, Psychology and Neuroscience; South London and Maudsley Mental Health NHS Trust
**Primary Subject Heading**:	Health informatics
Secondary Subject Heading:	Mental health
Keywords:	Child & adolescent psychiatry < PSYCHIATRY, Health informatics < BIOTECHNOLOGY & BIOINFORMATICS, World Wide Web technology < BIOTECHNOLOGY & BIOINFORMATICS

SCHOLARONE™
Manuscripts

I, the Submitting Author has the right to grant and does grant on behalf of all authors of the Work (as defined in the below author licence), an exclusive licence and/or a non-exclusive licence for contributions from authors who are: i) UK Crown employees; ii) where BMJ has agreed a CC-BY licence shall apply, and/or iii) in accordance with the terms applicable for US Federal Government officers or employees acting as part of their official duties; on a worldwide, perpetual, irrevocable, royalty-free basis to BMJ Publishing Group Ltd ("BMJ") its licensees and where the relevant Journal is co-owned by BMJ to the co-owners of the Journal, to publish the Work in this journal and any other BMJ products and to exploit all rights, as set out in our licence.

The Submitting Author accepts and understands that any supply made under these terms is made by BMJ to the Submitting Author unless you are acting as an employee on behalf of your employer or a postgraduate student of an affiliated institution which is paying any applicable article publishing charge ("APC") for Open Access articles. Where the Submitting Author wishes to make the Work available on an Open Access basis (and intends to pay the relevant APC), the terms of reuse of such Open Access shall be governed by a Creative Commons licence – details of these licences and which Creative Commons licence will apply to this Work are set out in our licence referred to above.

Other than as permitted in any relevant BMJ Author's Self Archiving Policies, I confirm this Work has not been accepted for publication elsewhere, is not being considered for publication elsewhere and does not duplicate material already published. I confirm all authors consent to publication of this Work and authorise the granting of this licence.

For peer review only

Investigating online activity in UK adolescent mental health patients: a feasibility study using a Natural Language Processing approach for Electronic Health Records

R Sedgwick[1,2], A Bittar[1], H Kalsi[1], T Barack[1], J Downs[1,2], *R Dutta[1,2]

[1]Institute of Psychiatry, Psychology and Neuroscience, King's College London

[2]South London and Maudsley NHS Foundation Trust

*Corresponding author: rina.dutta@kcl.ac.uk

For peer review only

ABSTRACT

Objectives: To assess the feasibility of using a Natural Language Processing (NLP) application for extraction of free text online activity mentions in adolescent mental health patient Electronic Health Records (EHRs).

Setting: The Clinical Records Interactive Search (CRIS) system allows detailed research based on de-identified EHRs from the South London and Maudsley NHS Foundation Trust (SLaM), a large south London Mental Health Trust providing secondary and tertiary mental health care.

Participants and Methods: We developed a gazetteer of online activity terms and annotation guidelines, from 5,480 clinical notes (200 adolescents, aged 11-17 years) receiving specialist mental health care. The pre-processing and manual curation steps of this real-world dataset allowed development of a rule-based NLP application to automate identification of online activity (internet, social media, online gaming) mentions in EHRs. The context of each mention was also recorded manually as: supportive, detrimental, or neutral in a subset of data for additional analysis.

Results: The NLP application performed with good precision (0.97) and recall (0.94) for identification of online activity mentions. Preliminary analyses found 34% of online activity mentions were considered to have been documented within a supportive context for the young person, 38% detrimental and 28% neutral.

Conclusion: Our results provide an important example of a rule-based NLP methodology to accurately identify online activity recording in EHRs, enabling researchers to now investigate associations with a range of adolescent mental health outcomes.

STRENGTHS AND LIMITATIONS OF THIS STUDY

- To the authors' knowledge this paper is the first of its kind to describe the feasibility and development of an NLP application for extraction of online activity mentions in EHRs for use in research.
- Recording of online activity in free text EHRs will be dependent on both patient report and the detail of documentation by clinicians, and therefore may not represent the full extent of young people's online use.

For peer review only - http://bmjopen.bmj.com/site/about/guidelines.xhtml

For peer review only

- Information extracted using the methods outlined in this paper could provide valuable avenues for further research into the recorded online activity of young adolescent mental health patients and associations with mental health outcomes.

BACKGROUND

Use of the internet, social media and online gaming are now ubiquitous amongst adolescents. There are general concerns about the potentially harmful impact of screentime on children and young adolescents health, and particularly their mental health [1]. There are also some more established, specific risks online, such as cyberbullying [2]. Internet use is associated with a wide range of adverse outcomes such as self-harm and suicidal behaviour [3], [4], disordered eating and body image issues [5], and symptoms of Attention Deficit Hyperactivity Disorder (ADHD) [6]. Problematic video-gaming and social media are also associated with several health issues, such as conduct problems and sedentary behavior [7]. In addition, there is growing evidence, beyond mental health research, for associations between technology and being overweight or obese [8], with poorer academic performance [9] and exacerbation of educational inequalities [10]. It is therefore imperative for mental health services to understand the role of online activity in the populations they serve.

Internet Gaming Disorder was added to the fifth Diagnostic Statistical Manual (DSM-5) [11] and Gaming Disorder added to the International Classification of Diseases (ICD-11) [12]. Age, gender, personality characteristics and parental behaviour may all influence adolescents' choice of games [13] and gaming can be done via a number of different devices, both online and offline. Digital platforms are commonly used by adolescents and a wealth of information may be shared online, providing opportunities for support, information, and education. There are now consensus recommendations that asking about online activities should be part of routine clinical assessments [14], [15], [16]. The prevalence with which these are noted in mental health assessments completed in Child and Adolescent Mental Health Services (CAMHS), and the context in which they are recorded, has not been studied to date. The existing evidence for the impact of online activity on adolescents is predominantly from cross-sectional survey data and often includes minimal detail about online activities, often with a focus on amount of use, or defined by terms such as Problematic Internet Use

For peer review only - http://bmjopen.bmj.com/site/about/guidelines.xhtml

(PIU) [17]. Given the wide range of social media platforms, devices, games, and content on the internet it will be important to gain a more nuanced and real-world understanding of what adolescents are engaging with online. Studying a clinical population of mental health patients will highlight which disorders may predispose adolescents to negative psychological and social impacts of online activity, but also what they find supportive. This study provides valuable contextualising information about the recording of online activity in clinical encounters with adolescent mental health patients.

There are validated measures for smartphone, internet and gaming addiction, the most commonly used being the Smartphone Addiction Scale (SAS) [18], Internet Addiction Test (IAT) and the Chen's Internet Addiction Scale (CIAS) [19], but these are not widely used by clinicians within the UK and there is significant heterogeneity within the research literature. As these structured scales are not commonly used in clinical practice, they will not be uploaded within structured fields on Electronic Health Records (EHRs). However, in the UK 83% of children aged 12-15 have a smartphone and 69% have at least one social media profile [20] and adolescents with mental disorders spend more time online than those without a mental disorder [21]. Adolescents may not show symptoms suggestive of behavioral addiction, but this does not mean that they are not engaging in activities that may be harmful.

CAMHS in the UK are usually accessed via primary care referral, or emergency services in the case of crisis presentations such as self-harm. The National Institute for Health and Care Excellence provides guidelines, and a framework for mental health care and assessment, but the EHR platform that this information is documented on varies between NHS trusts. As part of mental health assessment and follow-up, clinicians will often discuss the adolescent's interests and how they spend their time, as well as triggers to a recent episode or relapse, such as cyberbullying. The EHRs therefore contain unstructured free text data about online activity of adolescents in contact with CAMHS. Advances in health informatics mean that information extraction tools can be used to automate the extraction of such information.

Natural Language Processing (NLP) combines computational linguistics with machine learning to allow analysis of unstructured data. This approach has been used across a variety of clinical specialties and health providers to extract information on symptoms, with mental health as

For peer review only - http://bmjopen.bmj.com/site/about/guidelines.xhtml

one of the most prevalent target populations for study[22]. NLP has already created opportunities to analyse large textual datasets and can now accurately detect mentions of complex phenomena such as suicidality [23], [24], [25], [26] and obsessive compulsive symptoms [27]. This study seeks to answer the question of whether an NLP application can derive information on the similarly complex and broad construct of adolescent mental health patient online activity. This will have implication for researchers wishing to undertake large scale epidemiological research, as well as clinicians who could use this personalised data to inform patient care.

For peer review only

METHODS

Data Source

The Clinical Records Interactive Search (CRIS) system allows detailed research based on EHRs from the South London and Maudsley NHS Foundation Trust (SLaM), a large south London Mental Health Trust providing secondary and tertiary care to residents of Southwark, Lambeth, Lewisham and Croydon [28]. The use of CRIS data for research was approved by the Oxfordshire Research Ethics Committee C (reference 08/H0606/71 + 5). CRIS data is used by researchers in a de-identified and data-secure format and patients have the choice to opt-out of their data being used. CRIS approval for this project has been granted by the CRIS oversight committee (Project reference: 18-102) and all data for use in this research has been accessed in accordance with CRIS Governance procedures. Care may be provided in mental health settings such as clinics or psychiatric hospitals, or in acute health settings such as emergency departments. In 2014 there were 250,000 patient records[28]. As of September 2019, the EHRs of over 350,000 patients, including over 5.7 million text documents can be analysed. Clinicians may enter clinical information in a variety of different sections within the EHRs, including: events (unstructured notes), forms (i.e., risk assessment), or clinical document attachments such as letters. Events and letters are most commonly used to record clinical information and sometimes the same information may be duplicated across locations.

Clinical Cohort and Corpus Development

For peer review only - http://bmjopen.bmj.com/site/about/guidelines.xhtml

In order to develop an NLP application, it was necessary to generate an adolescent data set within CRIS. Event and attachment documents (n=1,601,422) were derived from 23,455 adolescent patients aged 11 to 17 in contact with CAMHS between 31/04/2009 and 31/03/2016, as described by Velupillai et al [29]. For the purpose of this paper n=number of documents, N=number of mentions of online activity. As illustrated in figure 1, from this, a corpus of documents was extracted from a randomly selected group of 200 patients who had a number of EHR documents within the 1st and 3rd quartiles (document n=5,480). This ensured that patients with particularly high or low numbers of records were excluded, as these patients were less likely to be representative of the general clinical population accessing CAMHS, either due to high intensity of contact (such as with prolonged inpatient care) or lack of contact due to non-engagement. Diagnosis was not used as an inclusion or exclusion criterion.

Mentions of online activity in EHRs

Key word searches were a basic but necessary first step to establish prevalence and variability of such terms within free text, especially for such a rapidly evolving and broad construct as online activity. Available literature was searched until there was a saturation of terms. The search included published work, grey literature publications online and policy documents. This was supported by clinical experience from within the research team and consultation with adolescents through face-to-face interactions at local patient advisory groups, including the Maudsley Biomedical Research Centre Young People's Mental Health Advisory Group (YPMHAG). This formed the basis of the gazetteer of terms, developed to convey topics that included online devices (i.e., computer, iPad), internet terms (e.g., websites, specific sites (e.g., YouTube), online games (e.g., Fortnite), social media terms (e.g., forum*) and specific platforms including Facebook, Twitter, and Instagram. The full gazetteer used for the final stages of this research is available in Table 1.

Table 1: Online activity gazetteer

**Social Media**	**Internet**	**Online Gaming**
#	Android	Call of Duty
4chan	Blackberry	Club Penguin

For peer review only - http://bmjopen.bmj.com/site/about/guidelines.xhtml

askFM	Computer	Computer gam*
Bebo	Dark Web	Computer-gam*
Blog*	Deep web	Coraline
Chatroom*	Googl*	Counter strike
cyber-bully*	Internet	Dota 2
cyberbully*	iphone	Dragon age
e-communi*	Laptop	Fallout
Face book	Mobile phone	Game Boy
Facebook	Online	Game-boy
FB	PC	Gaming
Flickr	Pinterest	Ghostbusters
Forum*	Skype	Grand Theft Auto
Hashtag*	Smartphone	HALO
Image Sharing	surf* the web	League of legends
Instagram	web address	Minecraft
Instant messag*	web brows*	Miniclip
Linkedin	web surfing	Nintendo
lolcow	web-brows*	Online Gam*
Myspace	web-surfing	PC gam*
Periscope	website*	Playstation
Recovery account	you tub*	PS3
Reddit	youtub*	PS4
Snapchat	ipad	Sims
Social Media	i-pad	Smite
Social Network*		video game
Spam Account		Wii
Tumblr		World of tanks
Tweet*		World of warcraft
Twitter		Xbox
Video sharing		X-box
Vimeo		Xmen
WhatsApp		X-men

For peer review only

Wordpress		Fortnite
		Pokemon
		Fortnight
		DS

Extracting EHRs for manual curation and pre-processing

The clinical corpus from the inception cohort of 200 patients was used for all subsequent analysis and development. Based on the rationale that a varied lexicon would be used to describe online media use, the gazetteer of key terms was used to identify and filter documents from the corpus with at least one of the search terms, to avoid reading a large volume of unrelated documents. By applying this filter, we identified 217 documents containing at least one of the terms, from 84/200 patients. These were used to gain further contextual insight and identify additional terms relevant to the concepts, including any common misspellings or abbreviations found (i.e., Face Book, FB). Documents with one or more terms from the gazetteer were analysed in detail by two researchers (RS and HK). Many documents were found to be irrelevant 'noise'. Examples were disclaimer messages at the bottom of email contacts, or use of the NHS Trust website in letter headers. The term 'email' was found to be generating too much noise for inclusion. Decisions such as this were agreed during regular consensus meetings with the research group.

Developing manual coding rules

To ensure that future research could be targeted towards more specific exposures it was necessary to split the search terms to represent three separate classes of mention: internet, social media, and online gaming. The class mention might refer to a specific social media platform or game from the gazetteer, or descriptive context, such as "playing games on the internet". Further details can be found in Appendix A. The manual curation also identified broad sentiment attributes within clinician documentation: Detrimental, Supportive or Neutral. For example, mention of Facebook in the context of bullying and a subsequent presentation to hospital would be coded as 'SOCIAL MEDIA_DETRIMENTAL'. During the initial scoping exercise supportive mentions were further split

For peer review only - http://bmjopen.bmj.com/site/about/guidelines.xhtml

into sub-categories to allow for more detailed future analysis and to better capture the context of mentions in the text. This included online activity that adolescents have referred to as supportive, clinician offered supportive advice (e.g., recommending online resources) and online activity which supports carers (e.g., use of a mental health support forum). Annotation guidelines were developed for the above class and attribute rules to facilitate consistent manual annotation by more than one researcher.

Manual annotation of Online Activity and sentiment attributes in EHRs

The pre-processing steps, when applied to the EHRs of the inception cohort, yielded a development corpus of 200 documents from the overall 5480 (derived from 89 of the 200 patients), which formed the dataset for the pilot analysis reported below in results. The corpus of 200 documents was divided and annotated for class and attributes by two researchers (RS and HK) using the annotation guidelines. Thirty documents were double annotated and there was an inter-annotator agreement of kappa coefficient=0.91 for class, 0.68 for attributes and 0.94 for supportive category [30].

Development of the Online Activity NLP application

The pre-processing steps outlined above paved the way for development of the NLP application, designed to automate identification of mentions of online activity use in EHRs. During the manual annotation (human-rater) stage, contextualising online activity raised some challenges. The sentiment attributes were found to be heterogeneous, often lacking detail and more subject to human inter-rater disagreement. Therefore, the algorithm was developed for automation of the class of mention only (internet, social media, or online gaming), based on the manual coding rules applied to the development corpus. Further details and examples can be found in the Annotation Guidelines, Appendix A.

The Online Activity NLP application is a rule-based system based on the spaCy NLP library for Python (version 2.1.3). The application uses four levels of processing, applied sequentially to each document:

For peer review only - http://bmjopen.bmj.com/site/about/guidelines.xhtml

1. *Text cleaning:* removal of "unwanted" document sections by regular expression replacement.
2. *Linguistic pre-processing:* sentence and word tokenisation, lemmatisation, and part-of-speech tagging.
3. *Lexical annotation:* terms in the text are tagged according to the gazetteer (e.g., 'computer', 'website' are tagged as INTERNET, 'cyberbully*', 'forum' and 'Instagram' are tagged as SOCIAL_MEDIA) and 'Fortnite' and 'online gaming' are tagged as ONLINE_GAMING.
4. *Token sequence annotation:* sequences of tokens (i.e., words) are annotated and classified (e.g., the pattern '(chat|communicat|talk)* online' is tagged as SOCIAL_MEDIA, '(play|playing) fortnite' is tagged as ONLINE_GAMING, etc. This step also removes annotations ("untags") from mentions that were erroneously tagged in the lexical annotation step.

Patient and public involvement

Development of the gazetteer of online activity terms was supported by face-to-face consultation with adolescent mental health patients through local patient advisory groups up to 2019, including presentation at the Maudsley Biomedical Research Centre YPMHAG.

RESULTS

The development corpus (n=200) documents extracted through the pre-processing steps (each document containing at least one term from the gazetteer) contained N=243 individual mentions of online activity. In some cases, the same information will be copied into different sections of EHRs but will appear as separate documents. These duplicate mentions, and others that were clearly irrelevant (i.e., relating to a typo) were removed (n=115). The remaining 101 documents (64 patients) contained 128 mentions of internet (N=64), social media (N=32), online gaming (N=32). Mean age was 14 (range 11-17 years), from 37 males and 27 females.

For peer review only

Contextualising mentions of online activity

There were in total, 44 supportive mentions (34%), 48 detrimental mentions (38%), 36 neutral mentions (28%). No 'other' mentions were recorded in this development corpus. Supportive mentions were sub-divided into supportive for the young person (N=25), where a clinician was offering supportive advice (N=17) or where a carer had reported an online activity as helpful (N=2). Each class was also analysed independently to provide pilot data on these different exposures. Internet mentions were 33% detrimental, 48% supportive, 19% neutral. Social media mentions were predominantly reported by female patients and classed as detrimental (50%), with little supportive benefits (9%). Online gaming was predominantly amongst male users and showed detrimental (34%), supportive (31%) and neutral (34%) context.

Evaluation of the Online Activity NLP application

An evaluation corpus was curated using EHRs from an expanded date range of the inception cohort, from CRIS origination to 02/07/2019. These adolescents were 11-17 at the time of presentation (between 2009-16), therefore it was anticipated that not all records (n= 12795) would be relevant. As the research group were interested in the CAMHS population specifically, only documents pertaining to adolescents who were still age 18 or younger at the time of documentation were included. Records of less than 50 words were removed due to the lack of relevance. Documents included in the development corpus were also removed (n=200). The remaining evaluation corpus (n=5972) was randomly divided between two researchers (RS and TB) and all relevant mentions of Internet/Social media/Online gaming were manually annotated according to the annotation guidelines (Appendix A). To establish the human inter-rater agreement, 200 documents, both with and without annotations were double annotated, yielding a kappa coefficient of 0.94 for annotation of class (internet, social media, online gaming). Sentiment attributes (detrimental, supportive, neutral) were again manually annotated, but as this process was not automated, these were not included in the evaluation. Following a consensus discussion, discrepancies were resolved. These predominantly related to mentions in older documents where limited detail was given for example "playing on the computer". Adjudicated documents were included to produce a 'gold standard' Evaluation Corpus containing 535 individual

For peer review only - http://bmjopen.bmj.com/site/about/guidelines.xhtml

annotations, from 5972 documents. This evaluation revealed a precision of 0.97 and recall of 0.94 and kappa=0.91. Span agreement showed a precision of 0.69 and recall 0.80, with full results available in Table 2.

Table 2: Performance of the Online Activity NLP application on the Evaluation Corpus (n=5972)

Evaluation results

	**Span agreement**	**Class (Internet, Social media, [illegible]**
**Precision (macro)**	0.69	0.97
**Recall (macro)**	0.80	0.94
**F-score (macro)**	0.74	0.95
**Precision (micro)**	N/A	0.95
**Recall (micro)**	N/A	0.95
**F-score (micro)**	N/A	0.95
**Kappa**	N/A	0.91

DISCUSSION

This study provides evidence of the feasibility of using free-text EHR data for the evaluation of online activity in sample of mental health patients and to the authors' knowledge is the first of its kind to use this methodology. The use of digital interventions in mental health is rapidly growing and there is interest in how these developments should be evaluated in future. In the meantime, it is vital that more evidence-based guidelines reach clinicians to ensure the quality of documentation facilitates research into this important and timely area. This is a move supported by the UK Royal College of Psychiatrists in their 2020 report, which calls for urgent funding of high-quality, longitudinal research into the effects of technology on the mental health of young people, and the need for technology companies to provide user-generated data for research [16].

For peer review only

For peer review only - http://bmjopen.bmj.com/site/about/guidelines.xhtml

Clinician mentions of online activity will be influenced by the clinician's personal knowledge and experience. The results of our scoping exercises revealed that despite guidance from the British Psychological Association [15] and Royal College of Psychiatrists [14] the detail of documentation has historically been poor. This is however likely to improve, given increasing acknowledgement of the important role of digital technology and mental health for adolescents. As more mental health applications and online resources are recommended by clinicians; familiarity will increase and these discussions will more frequently take place between patients and professionals at CAMHS. Subsequently, recording in free-text EHRs will improve and there may be scope for prospective data collection in the future, prompting clinicians to delineate further detail around online activity use.

The focus of NLP development within CRIS has historically been on symptoms, but detection of behaviours and activities adolescent engage in is also required if we are to better understand the impact on mental health. Automated detection of cyberbullying has been attempted in social media text [31], and a bullying NLP application has been developed [32]. The NLP application we outline here is important as it will be able to capture emerging online activities and behaviours in the EHRs of adolescents, providing opportunities for much needed longitudinal research into online activity and mental health and wellbeing, which to date has been lacking. These developments can inform our understanding of the specific risks and benefits of online exposures; inform clinical guidelines and help target future interventions caused by digital exposures and to evaluate interventions delivered through these platforms.

There are no similar studies available for direct comparison and therefore the strengths of the NLP application are encouraging. It has shown good precision (0.97) and recall (0.94) in automating detection of mentions of internet, social media, and online gaming in our corpus of clinical notes, enabling further research into online activities within a large adolescent mental health population. We divided online activity into broad classes: internet, social media, and online gaming, though it is worth noting that there is increasing overlap to these formats with technological advances. We found that broadly social media was reported in the EHRs as more harmful than online gaming, which may be an important hypothesis generating finding. The concepts of 'supportive', 'detrimental' and 'neutral', have been shown to be promising attributes for automation and incorporation into future iterations of

For peer review only - http://bmjopen.bmj.com/site/about/guidelines.xhtml

For peer review only

the online activity NLP application. However, limitations to human-coder agreement requires further work and a more nuanced taxonomy of terms may be required to accurately reflect the online activity of adolescents.

Detailed analysis about the generalisability of our findings to all mental health patients was outside the scope of this study. The use of unstructured retrospective EHR data has its limitations, in particular the potential for selection bias. Clinicians may have been more likely to document online activity for certain patient groups who they perceive to be more susceptible to detrimental or supportive impacts, or this may have been influenced by external factors such as publication of professional guidance or individual perception of the importance of these exposures to adolescent mental health. There are limitations to the clinical interpretation possible from our data. The pilot data reported in this paper included any mention of enjoyment as 'supportive' for the young person, unless there was any negating information. Identification and nurturing of enjoyable activities and hobbies can be a useful tool when working with adolescent patients in CAMHS. But, there is also the possibility of these becoming excessive and having a 'detrimental' impact, especially given the increasing concern about gaming disorder [11], [33]. Perspectives of the young person and a carer may differ, but this may not be documented by the clinician. It will also be necessary in later iterations of the NLP application to incorporate negating terms and phrases, as well as greater sensitivity for which subject (adolescents themselves or third party) the mention of online activity relates to. We found few (n=2) positive mentions of online activity by a parent or carer. Given that young people will be the focus of a clinical encounter, this likely reflects a lack of documentation regarding carer support. This is a limitation to our methodology, given that support and information for carers may increasingly be found online. This and other nuances will become increasingly important on more contemporary datasets, though it is worth noting that it was not considered a major limitation in the historical corpus reported here.

There are limitations to the rule-based approach with such a rapidly evolving field. We endeavoured to have a broad range of search terms in the gazetteer but acknowledge that this is not exhaustive. New games, social media platforms, websites, and apps are hard to keep up with and omission of these titles from the gazetteer may bias studies towards certain online activities. The

For peer review only - http://bmjopen.bmj.com/site/about/guidelines.xhtml

gazetteer can be added to and amended based on the context of the data and emergence of new popular terms, but this will require a degree of vigilance from users wishing to apply it to contemporary datasets, or those with other groups, such as adults or disorder-specific cohorts. The application displayed insufficient contextual disambiguation for the following words: computer, Internet, mobile phone, online, PC, website. It performed less well distinguishing class from longer spans of free text i.e., *playing games with friends online* or *playing games on the computer* being incorrectly labelled 'internet' rather than 'online gaming'. Mention of all specific websites described in CRIS would not be feasible, but inclusion of *www.,.co.uk* or other more generic identifiers resulted in too many false positives. Similarly, '*email*'* generated too many false positives during development to be included. These may therefore be false negatives that should be considered when using the NLP application and it is possible that in some circumstance's precision could be sacrificed for greater recall.

This paper outlines the developments in NLP for use in EHR's within CRIS, but the Online Activity NLP application could also be adapted for other clinical data sets to allow reproduction of results. User-generated data could be another application for this NLP approach and may more accurately capture adolescent online behaviour, unhampered by the recall and reporting bias associated with self-report questionnaires or discussion with a clinician. NLP can already be applied to risk assessment of self-injurious behaviour in user-generated content [34] and the Linguistic Inquiry and Word Count (LIWC) has shown promise in assessing emotional wellbeing from Facebook posts [35]. Social media data has potential as a rich data source for identification of medical and mental health conditions [36], [37] and with further refinement, discussion of risk-associated online activity or 'online harms' [38] could be another avenue for the application of NLP, such as that outlined here. These advancements could eventually lead to earlier detection of at-risk adolescents and targeting of interventions, a field already developing in suicide prevention [39]. There is also scope on a public health level for user generated content to be useful for communication, monitoring and prediction of disease, which was demonstrated during the COVID-19 pandemic [40].

There are also potential clinical applications for this work when applied to EHRs. The app could be valuable for characterising online activity patterns for specific patient groups, such as those

For peer review only - http://bmjopen.bmj.com/site/about/guidelines.xhtml

with eating disorders or Autism Spectrum Disorder, and the impact on later recovery. Since the Covid-19 pandemic, young people's online activity has accelerated, with greater reliance on online means of communication, education, and access to mental health support. Our NLP application could provide valuable insight into these trends; providing information on an individual and epidemiological level to guide recommendations. There is also potential for adaptation to more dynamic uses, such as EHR surveillance to track the burden of adverse online experiences through established methods such as Audit and feedback, which can result in important improvements in clinical practice[41]. Information such as this, presented in accessible ways such as clinician dashboards, could support rapid synthesis of risk factors within an individual or across a service and identify areas of unmet need and potential treatment targets.

CONCLUSION

We have developed a highly accurate Online Activity NLP application for use in EHRs, which can incorporate keywords as online platforms and services develop over time. This will allow further research using CRIS data to investigate novel risk factor research into a range of adolescent mental health outcomes. It also opens the door for EHR surveillance and clinical monitoring, enabling clinicians to track the burden of adverse online experiences at an individual and service level. Beyond its proven utilisation in EHRs, tools such as this also have the potential to be adapted to other clinical or non-clinical datasets, which could enhance our understanding of these new phenomena and the impact on adolescent health and wellbeing.

CONTRIBUTORSHIP STATEMENT

Rosemary Sedgwick developed the concept, and led on the study design, data collection, writing of results and final draft of the manuscript. Andre Bittar developed the NLP application and ran the evaluations. Manual coding rules were written by Rosemary Sedgwick with input from the other authors, including Johnny Downs and Rina Dutta. Reviewing of EHRs for the manual curation stage was performed by Rosemary Sedgwick and Herkiran Kalsi. Reviewing the EHRs for the Evaluation

For peer review only - http://bmjopen.bmj.com/site/about/guidelines.xhtml

Corpus was performed by Rosemary Sedgwick and Tamara Barack. All authors were involved in the writing and review of the manuscript. All authors declare no competing conflict of interest.

ACKNOWLEDGMENTS

The authors' acknowledge infrastructure support from the National Institute for Health Research (NIHR). The views expressed are those of the author(s) and not necessarily those of the NHS, the NIHR or the Department of Health and Social Care.

FUNDING STATEMENT

RS was funded by an National Institute for Health Research (NIHR) Academic Clinical Fellowship and a NIHR Biomedical Research Centre at South London and Maudsley NHS Foundation Trust Preparatory Fellowship.

JD is supported by an NIHR Clinician Science Fellowship award and has received support from a Medical Research Council (MRC) Clinical Research Training Fellowship and Psychiatry Research Trust Peggy Pollak Research Fellowship in Developmental Psychiatry.

RD is funded by a Clinician Scientist Fellowship from the Health Foundation in partnership with the Academy of Medical Sciences and her work is supported by the NIHR Biomedical Research Centre at South London and Maudsley NHS Foundation Trust and King's College London.

COMPETING INTERESTS

RD declares previous research funding received from Janssen.

The remaining authors declare that the research was conducted in the absence of any commercial or financial relationships that could be construed as a potential conflict of interest.

ETHICS APPROVAL

The use of CRIS data for research was approved by the Oxfordshire Research Ethics Committee C (reference 08/H0606/71 + 5). CRIS data is used by researchers in a de-identified and data-secure format and patients have the choice to opt-out of their data being used. CRIS approval for this project

For peer review only

For peer review only - http://bmjopen.bmj.com/site/about/guidelines.xhtml

has been granted by the CRIS oversight committee (Project reference: 18-102) and all data for use in this research has been accessed in accordance with CRIS Governance procedures.

DATA SHARING

The data accessed by CRIS remain within an NHS firewall and governance is provided by a patient-led oversight committee. Access to data is restricted to honorary or substantive employees of the South London and Maudsley NHS Foundation Trust and governed by a local oversight committee who review and approve applications to extract and analyse data for research. Subject to these conditions, data access is encouraged and those interested should contact the CRIS academic lead.

REFERENCES

1. Stiglic N, Viner RM. Effects of screentime on the health and well-being of children and adolescents: a systematic review of reviews. BMJ Open. 2019;9(1):e023191.
2. John A, Glendenning AC, Marchant A, Montgomery P, Stewart A, Wood S, et al. Self-Harm, Suicidal Behaviours, and Cyberbullying in Children and Young People: Systematic Review. Journal of Medical Internet Research. 2018;20(4):e129.
3. Marchant A, Hawton K, Stewart A, Montgomery P, Singaravelu V, Lloyd K, et al. A systematic review of the relationship between internet use, self-harm and suicidal behaviour in young people: The good, the bad and the unknown. PLoS One. 2017;12(8):e0181722.
4. Sedgwick R, Epstein S, Dutta R, Ougrin D. Social media, internet use and suicide attempts in adolescents. LID - 10.1097/YCO.0000000000000547 [doi]. Curr Opin Psychiatry. 2019(1473-6578 (Electronic)).
5. Holland G, Tiggemann M. A systematic review of the impact of the use of social networking sites on body image and disordered eating outcomes. Body Image. 2016;17:100-10.
6. Ra CK, Cho J, Stone MD, De La Cerda J, Goldenson NI, Moroney E, et al. Association of Digital Media Use With Subsequent Symptoms of Attention-Deficit/Hyperactivity Disorder Among Adolescents. JAMA. 2018;320(3):255-63.
7. Merelle SYM, Kleiboer AM, Schotanus M, Cluitmans TLM, Waardenburg CM, Kramer D, et al. Which health-related problems are associated with problematic video-gaming or social media use in adolescents? A large-scale cross-sectional study. Clinical Neuropsychiatry. 2017;14(1):11-9.
8. Herodotou C. Young children and tablets: A systematic review of effects on learning and development. Journal of Computer Assisted Learning. 2018;34(1):1-9.
9. Sampasa-Kanyinga H, Chaput J-P, Hamilton HA. Social Media Use, School Connectedness, and Academic Performance Among Adolescents. The Journal of Primary Prevention. 2019;40(2):189-211.
10. Performance CfE. Ill Communication: Technology, distraction & student performance Discussion Paper No 1350. 2015.
11. Arlington V. Diagnostic and Statistical Manual of Mental Disorders, Fifth Edition.: American Psychiatric Association; 2013.
12. Office for national statistics. 2018. Internet access- households and individuals: 2018 2018 [Available from:

For peer review only - http://bmjopen.bmj.com/site/about/guidelines.xhtml

https://www.ons.gov.uk/peoplepopulationandcommunity/householdcharacteristics/homeinternetandsocialmediausage/bulletins/internetaccesshouseholdsandindividuals/2018.
13. John N, Sharma MK, Kapanee ARM. Gaming- a bane or a boon-a systematic review. Asian J Psychiatry. 2019;42:12-7.
14. Psychiatrists RCo. Child & Adolescent Psychiatry Curriculum: January 2018 amendments. 2018.
15. Bristow F, Roberts A. Top tips for working with children, young people and their families: Supporting trainees and other pre-qualified clinicians. British Psychological Society. 2015.
16. RCPsych. Technology use and the mental health of children and young people. 2020;College Report CR225.
17. Spada MM. An overview of problematic Internet use. Addictive Behaviors. 2014;39(1):3-6.
18. Kwon M, Kim D-J, Cho H, Yang S. The Smartphone Addiction Scale: Development and Validation of a Short Version for Adolescents. PLOS ONE. 2014;8(12):e83558.
19. Costa S, Kuss DJ. Current diagnostic procedures and interventions for Gaming Disorders: A Systematic Review. Front Psychol. 2019;10:578-.
20. Ofcom. Children and Parents: Media Use and Attitudes Report 2018. Ofcom; 2019.
21. Digital N. Mental Health of Children and Young People in England, 2017. 2018.
22. Koleck TA, Dreisbach C, Bourne PE, Bakken S. Natural language processing of symptoms documented in free-text narratives of electronic health records: a systematic review. J Am Med Inform Assoc. 2019;26(4):364-79.
23. Polling C, Tulloch A, Banerjee S, Cross S, Dutta R, Wood DM, et al. Using routine clinical and administrative data to produce a dataset of attendances at Emergency Departments following self-harm. BMC Emerg Med. 2015;15(15):015-0041.
24. Fernandes AC, Dutta R, Velupillai S, Sanyal J, Stewart R, Chandran D. Identifying Suicide Ideation and Suicidal Attempts in a Psychiatric Clinical Research Database using Natural Language Processing. Scientific Reports. 2018;8:7426.
25. Downs J, Velupillai S, Gkotsis G, Holden R, Kikoler M, Dean H, et al. Detection of Suicidality in Adolescents with Autism Spectrum Disorders: Developing a Natural Language Processing Approach for Use in Electronic Health Records. AMIA Annual Symposium Proceedings. 2017:641-9.
26. Velupillai S, Hadlaczky G, Baca-Garcia E, Gorrell GM, Werbeloff N, Nguyen D, et al. Risk Assessment Tools and Data-Driven Approaches for Predicting and Preventing Suicidal Behavior. Frontiers in Psychiatry. 2019(1664-0640 (Print)).
27. Chandran D, Robbins DA, Chang C-K, Shetty H, Sanyal J, Downs J, et al. Use of Natural Language Processing to identify Obsessive Compulsive Symptoms in patients with schizophrenia, schizoaffective disorder or bipolar disorder. Scientific Reports. 2019;9(1):14146.
28. Perera G, Broadbent M, Callard F, Chang C-K, Downs J, Dutta R, et al. Cohort profile of the South London and Maudsley NHS Foundation Trust Biomedical Research Centre (SLaM BRC) Case Register: current status and recent enhancement of an Electronic Mental Health Record-derived data resource. BMJ Open. 2016;6(3):e008721.
29. Velupillai S, Epstein S, Bittar A, Stephenson T, Dutta R, Downs J. Identifying Suicidal Adolescents from Mental Health Records Using Natural Language Processing. Studies in health technology and informatics. 2019;264:413-7.
30. Cohen J. A Coefficient of Agreement for Nominal Scales. Educational and Psychological Measurement. 1960;20(1):37-46.
31. Van Hee C, Jacobs G, Emmery C, Desmet B, Lefever E, Verhoeven B, et al. Automatic detection of cyberbullying in social media text. PloS one. 2018;13(10):e0203794-e.
32. Holden RM, Joanne; Mcgowan, John; Sanyal, Jyoti, Kikoler, Maxim; Simonoff, Emily; Velupillai, Sumithra ; Downs, Johnny. Investigating Bullying as a predictor of Suicidality in a Clinical Sample of Adolescents with Autism Spectrum Disorder. Autism Research. 2020.
33. International statistical classification of diseases and related health problems (11th Revision): World Health Organization; 2018 [Available from: https://icd.who.int/browse11/l-m/en#/http://id.who.int/icd/entity/1448597234.
34. Franz PJ, Nook EC, Mair P, Nock MK. Using Topic Modeling to Detect and Describe Self-Injurious and Related Content on a Large-Scale Digital Platform. LID - 10.1111/sltb.12569 [doi]. Suicide & life-threatening behavior. 2019(1943-278X (Electronic)).

For peer review only - http://bmjopen.bmj.com/site/about/guidelines.xhtml

35. Settanni M, Marengo D. Sharing feelings online: studying emotional well-being via automated text analysis of Facebook posts. Front Psychol. 2015;6:1045-.
36. Thorstad R, Wolff P. Predicting future mental illness from social media: A big-data approach. Behav Res Methods. 2019;51(4):1586-600.
37. Merchant RMA-Ohoo, Asch DA, Crutchley PA-Ohoo, Ungar LH, Guntuku SCA-Ohoo, Eichstaedt JC, et al. Evaluating the predictability of medical conditions from social media posts. PLoS One. 2019;14(6).
38. Government H. Online Harms White Paper. 2019.
39. Lopez-Castroman JA-Ohoo, Moulahi B, Aze J, Bringay S, Deninotti J, Guillaume S, et al. Mining social networks to improve suicide prevention: A scoping review. LID - 10.1002/jnr.24404 [doi]. Journal of Neuroscience Research. 2019(1097-4547 (Electronic)).
40. Gunasekeran DA-O, Chew AA-O, Chandrasekar EA-O, Rajendram PA-O, Kandarpa VA-O, Rajendram MA-O, et al. The Impact and Applications of Social Media Platforms for Public Health Responses Before and During the COVID-19 Pandemic: Systematic Literature Review. J Med Internal Res. 2022;24(4):e33680.
41. Ivers N, Jamtvedt G Fau - Flottorp S, Flottorp S Fau - Young JM, Young Jm Fau - Odgaard-Jensen J, Odgaard-Jensen J Fau - French SD, French Sd Fau - O'Brien MA, et al. Audit and feedback: effects on professional practice and healthcare outcomes. Cochrane Database Syst Rev. 2012;6(1469-493X (Electronic)).

FIGURE LEGENDS

Figure 1: Method for developing a rule-based NLP application for Online Activity. n= number of documents. N=number of mentions.

For peer review only

Figure 1: Method for developing a rule-based NLP application for Online Activity. n= number of documents. N=number of mentions.

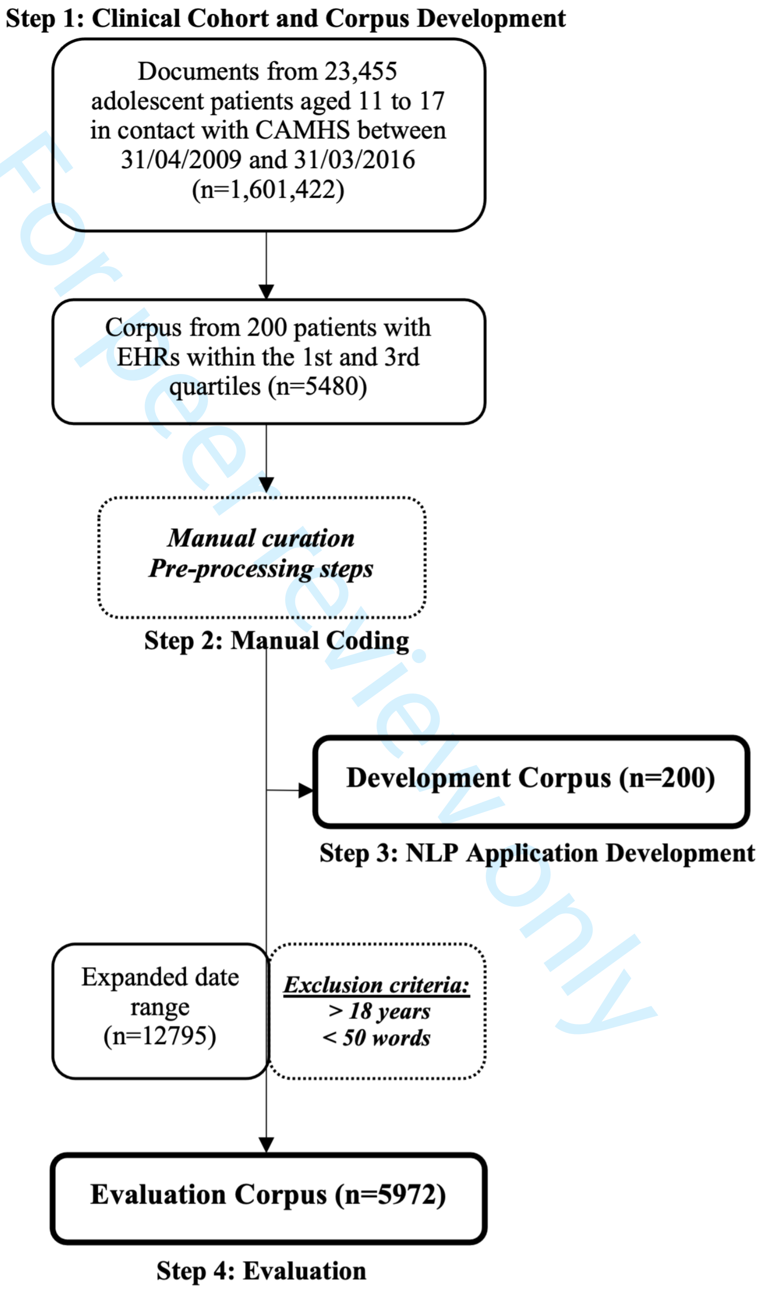

For peer review only

APPENDIX A: Annotation guidelines for adolescents Online Activity in CRIS

Introduction

This document contains the annotation guidelines for annotating clinician mentions of online activity in clinical text. We have broadly grouped online activity into groups: social media, internet, and online gaming, though there is some overlap. The aim is to be able to automatically identify clinician mentions of these factors documented in mental health records to then investigate associations between these exposures and self-harm outcomes in adolescents.

General Annotating

- You will need to read each document from start to finish for relevant mentions; which will then be highlighted.
- Where possible annotate only the relevant word(s).
- In some cases, a larger section of annotation will be required- annotate as much as is required to give context.
- All annotations should be given a class and an attribute from the below options.
- Each individual annotation can have only one of each.
- Not all mentions are explicit, we will accept inferred mentions if you can ascertain meaning from the context, further guidance below.

SOCIAL MEDIA<br>INTERNET<br>ONLINE GAMING

Class Annotating

Social Media

We are interested in patterns and the nature of social media use. Social media is for these guidelines defined as: *websites and applications that enable users to create and share content or to participate in social networking.* Mentions may refer to specific platforms included in the gazetteer such as: Instagram, Twitter, Facebook, Snapchat, or to a behaviours.

Examples: "Chatting to their friends online", "Talking to friends online"

Internet

We are interested in patterns and the nature of internet use and content viewed or shared online.

Examples:"... spends a lot of time online"

Online Gaming

We are interested in online gaming and have included general terms and more specific titles of games commonly used within the timeframe of our dataset.

Example: "Spends a lot of time playing video games"
Example: "Playing games on the internet"

Other online use

Since social media and internet activity are often accessed via mobile devices we have also included: *iPhone*, *iPad*, *Blackberry*, *Smartphone*. Where there is suggestion that these are used for online gaming or social media annotate accordingly if the exact use is not clear annotate as INTERNET.

For peer review only - http://bmjopen.bmj.com/site/about/guidelines.xhtml

For peer review only

Specific platforms

Pinterest
YouTube

These can have social media-like functions but are more commonly viewed as internet platforms and should therefore be annotated as INTERNET.

Attribute Annotating

Classification:<br>DETRIMENTAL<br>NEUTRAL<br>OTHER<br><br>Supportive_Category:<br>OTHER<br>SUPPORTIVE_CARER<br>SUPPORTIVE_PROFESSIONAL_ADVICE<br>SUPPORTIVE_YOUNG_PERSON

Detrimental

This attribute is for mentions which appear to be having a negative impact on the young person, either directly (such as via bullying) or indirectly (restricting other activities, causing arguments, affecting sleep).

Examples:
"playing computer games most of the time rather than continuing with his work"
"sleep is disturbed as z spends a great deal of time at home on the computer"
"received a message on Facebook from her best friend saying that their friendship was fake"

Neutral

Neutral attributes are where it is not possible from the text to determine the context or sufficient detail to ascribe a detrimental or supportive label.

Examples:
"does not go on Facebook or chat rooms"
"z is spending more time on the computer"

Supportive

SUPPORTIVE_CARER

This attribute is used for mentions which appear to relate to advice or support that the carer is receiving, likely in relation to the young person's condition.

Example: "mum reports finding the National Autistic Society parent forum a helpful resource"

SUPPORTIVE_PROFESSIONAL_ADVICE

This would be relevant if a clinician has recommended or signposted the young person or carer to resources online.

Example: "I gave mum the details of the National Autistic Society website"

SUPPORTIVE_YOUNG_PERSON

This could be because a digital resource has been specifically helpful. In addition, we classify mentions of a child 'liking' or 'enjoying' am activity as supportive. The rationale for this is that for young people with mental health difficulties enjoyable activities are often incorporated therapeutically.

For peer review only - http://bmjopen.bmj.com/site/about/guidelines.xhtml

Example: "z and his friend are making and editing Minecraft videos to upload to YouTube and he reports that this makes him feel happy".
Example: "z found a useful anti-bullying website"
Example: "z relaxes watching videos on YoutTube"

Additional refinements

When annotating highlight as much of the sentence/paragraph required to understand the context of the mention.

If it is clear that a document is a copy/paste version of one in the same corpus (this may be that an event has been copied to create a letter, or subtly different versions of the same letter have been sent to different parties) these should be annotated consistently.

Ambiguous examples:

Sometimes there are mentions which are ambiguous or appear to suggest contradictory qualities.

If context was creating a website this could be internet (i.e. YouTube), if for social networking site/sharing with contacts (i.e. Instagram) might be social media.

Example: "...posting images/photos online"

With no additional context we do not know what exact activity this is referring to (could be non-web-based, web browsing, online gaming, or social media).

Example: " enjoys playing on the computer"
Example: "He loves playing on the computer, but mum is worried that this stopping him socialising or doing normal activities with the family"

Clusters of mentions

Two mentions of different classes written one after the other can be coded separately.

*Example: "she likes playing **video games** and watching **videos online**".*
video games ONLINE GAMING>SUPPORTIVE_YP
videos online INTERNET>SUPPORTIVE_YP.

*Example: "Spends a lot of time playing **video games** like **Fortnite**"*
video games ONLINE GAMING>NEUTRAL
fortnite ONLINE GAMING>NEUTRAL

*Example: "She is being bullied via **Facebook, Instagram and Snapchat**"*
Facebook SOCIAL MEDIA>DETRIMENTAL
Instagram SOCIAL MEDIA>DETRIMENTAL
Snapchat SOCIAL MEDIA>DETRIMENTAL

*Example**: "**Z loves to read, watch movies or TV clips on **YouTube** and play **Sims"***
YouTube INTERNET>SUPPORTIVE_YP
Sims ONLINE GAMING>SUPPORTIVE_YP

Negated mentions

Negation detection

"...does not use social media"
"...has not been bullied on Facebook"
"...avoiding the internet as this was not helpful and adding stress"
"...now playing video games less"

For peer review only - http://bmjopen.bmj.com/site/about/guidelines.xhtml
